# Abstracts from the 2018 Respiratory Effectiveness Group Summit: Amsterdam, the Netherlands. 22-24 March 2018

**DOI:** 10.1186/s12931-018-0930-9

**Published:** 2018-12-20

## REGABS18001: Withdrawn

## REGABS18002: Withdrawn

## REGABS18003: A novel observational longitudinal study in patients with asthma and/or COPD: NOVELTY design, objectives and analyses

### Maria Gerhardsson de Verdier^1^, Alvar Agustí^2^, Gary P Anderson^3^, Aruna T Bansal^4^, Richard Beasley^5^, Elisabeth H Bel^6^, Christer Janson^7^, Barry Make^8^, Ian Pavord^9^, David Price^10^, Lance Brannman^11^, Donna K Finch^12^, Asparuh Gardev^13^, Niklas Karlsson^1^, Christina Keen^14^, Javier Nuevo^15^, Stephen Rennard^16^, Helen K Reddel^17^

#### ^1^AstraZeneca, Gothenburg, Sweden; ^2^Respiratory Institute, Hospital Clinic, University of Barcelona, IDIBAPS, CIBERES, Barcelona, Spain; ^3^Lung Health Research Centre, University of Melbourne, Melbourne, VIC, Australia; ^4^Acclarogen Ltd, Cambridge, UK; ^5^Medical Research Institute of New Zealand, Wellington, New Zealand; ^6^Academic Medical Center, University of Amsterdam, Amsterdam, The Netherlands; ^7^Department of Medical Sciences, Uppsala University, Uppsala, Sweden; ^8^National Jewish Health and University of Colorado Denver, Denver, CO, USA; ^9^University of Oxford, Oxford, UK; ^10^Observational and Pragmatic Research Institute, Singapore and Centre of Academic Primary Care, University of Aberdeen, Aberdeen, UK; ^11^AstraZeneca, Gaithersburg, MD, USA; ^12^MedImmune Ltd., Cambridge, UK; ^13^AstraZeneca, Cambridge, UK; ^14^Respiratory, Inflammation and Autoimmunity, IMED Biotech Unit, AstraZeneca, Gothenburg, Sweden; ^15^AstraZeneca, Madrid, Spain; ^16^Early Clinical Development, IMED Biotech Unit, AstraZeneca, Cambridge, UK; ^17^Woolcock Institute of Medical Research, University of Sydney, Sydney, Australia;

**Background:** Asthma and chronic obstructive pulmonary disease (COPD) have traditionally been viewed as distinct disorders. Past studies have often focused on specific aspects of each disease based on a single diagnostic label, with strict clinical trial enrolment criteria and limited generalisability. There are few prospective, observational studies that include patients across asthma, COPD and asthma-COPD overlap diagnoses. Consequently, the ways in which these different clinical profiles relate to specific phenotypes and endotypes are not well understood. NOVELTY (a NOVEL observational longiTudinal studY in patients with asthma and/or COPD; clinicaltrials.gov NCT02760329) aims to describe patient characteristics, treatment patterns and disease burden across the spectrum of asthma and COPD over time, and to identify phenotypes and molecular endotypes associated with differential outcomes for symptom burden, clinical evolution and healthcare utilisation (HCU).

**Method:** NOVELTY is a global, prospective, observational, longitudinal cohort study that will include up to 12,000 patients ≥12 years of age with a diagnosis or suspected diagnosis of asthma and/or COPD across the spectrum of severities (balanced across diagnoses and severities), from primary and specialist care. The study will collect data on clinical assessments, spirometry, biospecimens (blood and urine), patient-reported outcomes (PROs) and HCU at baseline and longitudinally for 3 years. Data captured at annual visits will be recorded in study-specific electronic case report forms, with some PROs and HCU data collected remotely every 3 months. Recruitment is expected to end in March 2018 (except in China), followed by baseline analysis.

**Results:** NOVELTY will provide a rich data source that may enable novel classification of patients across the spectrum of obstructive lung disease according to clinical outcomes, PROs and biomarker profiles over time. It will create a biobank and data platform of evidence from clinical practice, which can also be utilised in the future by the broader community of physicians, patients, regulators and the scientific community.

**Conclusion:** Results from NOVELTY will provide insights into the diagnosis, assessment and management of patients with asthma and/or COPD in primary and specialist care around the world and may help support the future development of novel therapies and personalised healthcare in obstructive lung disease. The data platform created by NOVELTY will also be a valuable resource for research beyond the core objectives of the study itself.

**Disclosures:** MGdV, LB, AG, NK, CK, JN, SR are employees of AstraZeneca (AZ); DKF is an employee of an AZ Group company.

AA: speaking, participating in advisory boards and/or research from AZ, Chiesi, Menarini, Novartis (Nov) and Teva.

GPA: honoraria and advisory boards for AZ, Boehringer Ingelheim (BI), GlaxoSmithKline (GSK), Nov and Pieris.

ATB: paid consultant of AZ.

RB: research funding from AZ, Genentech, GSK and Health Research Council of New Zealand; lectures and/or participation in advisory boards from AZ, GSK and Health Research Council of New Zealand.

EHB: research grants from AZ, GSK and Nov; research consulting from AZ, BI, GSK, Nov, Sanofi/Regeneron, Teva and Vectura.

CJ: speaking and/or participating in advisory boards for AZ, BI, GSK, Nov and Teva.

BM: participation in NOVELTY planning meetings for AZ and in medical advisory boards for AZ, BI, CSL Bering, GSK, Nov, Spiration, Sunovion and Verona; research studies funded by AZ, BI, GSK, Medscape, Pearl and Sunovion; grant funding from AZ; non-branded talk for AZ.

IP: speaking at sponsored meetings from Aerocrine, Almirall, AZ, BI, GSK and Nov; attending advisory panels with Almirall, AZ, BI, Dey, Genentech, GSK, MSD, Napp, Nov, Regeneron, Respivert and Schering-Plough; organising an educational event from AZ; attending scientific meetings from AZ, BI, GSK and Napp.

DP: board membership with Aerocrine, Amgen, AZ, BI, Chiesi, Meda, Mundipharma (Mund), Napp, Nov, and Teva; consultancy agreements with Almirall, Amgen, AZ, BI, Chiesi, GSK, Meda, Mund, Napp, Nov, Pfizer, Teva, and Theravance; grants and unrestricted funding for investigator-initiated studies (conducted through Observational and Pragmatic Research Institute Pte Ltd) from Aerocrine, AKL Ltd, AZ, BI, British Lung Foundation, Chiesi, Meda, Mund, Napp, Nov, Pfizer, Respiratory Effectiveness Group, Takeda, Teva, Theravance, UK National Health Service, Zentiva; payment for lectures/speaking engagements from Almirall, AZ, BI, Chiesi, Cipla, GSK, Kyorin, Meda, Merck, Mund, Nov, Pfizer, Skyepharma, Takeda, and Teva; payment for manuscript preparation from Mund and Teva; payment for the development of educational materials from Mund and Nov; payment for travel/accommodation/meeting expenses from Aerocrine, AZ, BI, Mund, Napp, Nov, and Teva; funding for patient enrolment or completion of research from Chiesi, Nov, Teva, and Zentiva; stock/stock options from AKL Ltd which produces phytopharmaceuticals; owns 74% of the social enterprise Optimum Patient Care Ltd, UK and 74% of Observational and Pragmatic Research Institute Pte Ltd, Singapore; and is peer reviewer for grant committees of the Efficacy and Mechanism Evaluation programme, HTA, and Medical Research Council.

HKR: advisory boards for AZ, GSK and Nov; data safety monitoring boards for AZ, GSK, Merck and Nov; independent educational presentations for AZ, BI, GSK, Mund, Nov and Teva; and independent research funding from AZ and GSK.

## REGABS18004: Withdrawn

## REGABS18005: Withdrawn

## REGABS18006: Withdrawn

## REGABS18007: Withdrawn

## REGABS18008: Withdrawn

## REGABS18009: Withdrawn

## REGABS18010: Demographic and clinical characteristics of patients with severe asthma worldwide

### UK Severe Asthma Registry, Severe Asthma Network Italy, Severe Asthma Web-based Database, Korean Academy of Asthma, Allergy and Clinical Immunology, National Jewish Health, Trung Tran^1^, David Price^2,3,4^

#### ^1^AstraZeneca, Gaithersburg, MD, USA; ^2^Optimum Patient Care, Cambridge, UK; ^3^Observational and Pragmatic Research Institute, Singapore, Singapore; ^4^Academic Primary Care, University of Aberdeen, Aberdeen, UK; ^5^UK Severe Asthma Registry, Queen’s University Belfast, Belfast, Northern Ireland; ^6^UK Severe Asthma Registry, Barts Health NHS Trust, London, UK; ^7^UK Severe Asthma Registry, Guy's and St Thomas' NHS Trust, London, UK; ^8^UK Severe Asthma Registry, Royal Brompton & Harefield NHS Foundation Trust, London, UK; ^9^Personalized Medicine Asthma & Allergy Clinic, Humanitas University & Research Hospital, Milan, Italy; ^10^Severe Asthma Network Italy, Milan, Italy; ^11^Australasian Severe Asthma Network, Priority Research Centre for Healthy Lungs, University of Newcastle, Newcastle, Australia; ^12^Hunter Medical Research Institute, Department of Respiratory and Sleep Medicine, John Hunter Hospital, Newcastle, New South Wales, Australia; ^13^Catholic University of Korea, Seoul, South Korea; ^14^Division of Allergy and Clinical Immunology, Department of Internal Medicine, Asan Medical Center, University of Ulsan College of Medicine, Seoul, Korea; ^15^National Jewish Health, Denver, Colorado, USA

Collaborators:

UK Severe Asthma Registry: Liam Heaney^5^, John Busby^5^, Paul Pfeffer^6^, David Jackson^7^, Andrew Menzies-Gow^8^;

Severe Asthma Network Italy: G. Walter Canonica^9^, Enrico Heffler^9^, Concetta Sirena^10^;

Severe

Asthma Web-based Database: Peter Gibson^11,12^, Erin Harvey^11,12^, Heather Powell^12^;

Korean Academy of Asthma, Allergy and Clinical Immunology: Chin Kook Rhee^13^, You Sook Cho^14^;

National Jewish Health: Eileen Wang^15^, Pearlanne Zelarney^15^

**Background:** The lack of a universally accepted definition for severe asthma hinders the investigation into its exact prevalence and pathology. The International Severe Asthma Registry (ISAR) was created as a global effort to capture information on severe asthma using a standardized method of data capture. We aimed to examine the global prevalence of severe asthma and its corresponding patient characteristics.

**Method:** This was a descriptive study utilizing patients with severe asthma data recorded in the ISAR from the UK, USA, Italy, Australia and South Korea from December 2014 to December 2017. Patients were included in the ISAR if they were ≥18 years of age and were on GINA (Global Initiatives for Asthma) Step 5 therapy or Step 4 with uncontrolled symptoms. Descriptive statistics for demographic factors and clinical characteristics were tabulated and summarized.

**Results**: From a total of 2,244 patients with severe asthma, 1,502 (66.9%) patients were classified as GINA Step 5 patients and 742 (33.1%) as GINA Step 4 patients with uncontrolled symptoms. From the total study population, 1,250 (55.7%) were females and 1,120 (49.9%) were of Caucasian origin. Most of the patients were between the ages 55 and 79 (1107 (49.3%)) and were non-smokers (1,468 (66.2%)). A significant proportion (602 (49.9%)) of the patients had poorly controlled asthma. The asthma age of onset for Step 4 patients fell predominantly within the “>40” age category (291 (41.9%)), whereas the majority of Step 5 patients’ asthma age of onset fell within the “12-40” age category (268, (46.4%)). The most prevalent comorbidity was allergic rhinitis for Step 4 (317 (52.7%)) and Step 5 patients (329 (27.3%)). Blood eosinophil count was greater than 0.3 10^9^/L for 319 (48.9%) Step 4 and 911 (63.8%) Step 5 patients. Intermediate (25-50 parts per billion) or high (>50 parts per billion) Fractional Exhaled Nitric Oxide (FeNO) results were recorded for 1,107 (78.6%) patients while 1,307 (69.3%) patients had serum IgE levels within 150-400 IU/ml or above 400 IU/ml, indicative of pulmonary inflammation. At least one exacerbation was reported for 962 (78.4%) patients and 472 (49.1%) of these patients had a minimum of four or more exacerbations.

**Conclusion:** The demographic and clinical characteristics of patients with severe asthma from five geographically diverse countries support previously reported characteristics of severe asthma patients. To decipher informative trends in asthma phenotypes and clinical management, country-specific distributions should be compared next.

**Disclosures:** Liam Heaney has taken part in advisory boards and given lectures at meetings supported by GlaxoSmithKline, Respivert, Merck Sharpe & Dohme, Nycomed, Boehringer Ingelheim, Teva, Vectura, Novartis and AstraZeneca. He declares sponsorship for attending international scientific meetings from AstraZeneca, Boehringer Ingelheim, GlaxoSmithKline and Napp Pharmaceuticals; and speaker fees from AstraZeneca, Aerocrine, Hoffman la Roche and Teva.

Paul Pfeffer has taken part in advisory boards and given lectures at meetings supported by Novartis, GlaxoSmithKline, Boehringer Ingelheim, and AstraZeneca.

Andrew Menzies-Gow declares grants from AstraZeneca, Boehringer Ingelheim, GlaxoSmithKline and Hoffman La Roche; consultancy agreements with AstraZeneca; personal fees from AstraZeneca, Boehringer Ingelheim, GlaxoSmithKline, Hoffman La Roche, Napp Pharmaceuticals, Novartis, Teva and Vectura; non-financial support from AstraZeneca, Boehringer Ingelheim and Napp Pharmaceuticals; and payment for travel expenses to international conferences from AstraZeneca, Boehringer Ingelheim and Napp Pharmaceuticals.

Peter Gibson declares grants from AstraZeneca, GlaxoSmithKline and Novartis; and personal fees from AstraZeneca and GlaxoSmithKline.

Chin Kook Rhee declares consultancy and lecture fees from AstraZeneca, Boehringer Ingelheim, GlaxoSmithKline, MSD, Mundipharma, Novartis, Sandoz, Takeda and Teva-Handok.

Trung Tran is an employee of AstraZeneca.

David Price declares board membership with Aerocrine, Amgen, AstraZeneca, Boehringer Ingelheim, Chiesi, Mylan, Mundipharma, Napp Pharmaceuticals, Novartis and Teva; consultancy agreements with Almirall, Amgen, AstraZeneca, Boehringer Ingelheim, Chiesi, GlaxoSmithKline, Mylan, Mundipharma, Napp Pharmaceuticals, Novartis, Pfizer, Teva and Theravance; grants and unrestricted funding for investigator-initiated studies (conducted through Observational and Pragmatic Research Institute Pte Ltd) from Aerocrine, AKL Research and Development Ltd, AstraZeneca, Boehringer Ingelheim, British Lung Foundation, Chiesi, Mylan, Mundipharma, Napp Pharmaceuticals, Novartis, Pfizer, Respiratory Effectiveness Group, Teva, Theravance, UK National Health Service and Zentiva; payment for lectures/speaking engagements from Almirall, AstraZeneca, Boehringer Ingelheim, Chiesi, Cipla, GlaxoSmithKline, Kyorin, Mylan, Merck, Mundipharma, Novartis, Pfizer, Skyepharma and Teva; payment for manuscript preparation from Mundipharma and Teva; payment for the development of educational materials from Mundipharma and Novartis; payment for travel/accommodation/meeting expenses from Aerocrine, AstraZeneca, Boehringer Ingelheim, Mundipharma, Napp Pharmaceuticals, Novartis and Teva; funding for patient enrolment or completion of research from Chiesi, Novartis, Teva and Zentiva; stock/stock options from AKL Research and Development Ltd which produces phytopharmaceuticals; owns 74% of the social enterprise Optimum Patient Care Ltd (Australia, Singapore, and UK) and 74% of Observational and Pragmatic Research Institute Pte Ltd (Singapore); and is a peer reviewer for grant committees of the Efficacy and Mechanism Evaluation programme and Health Technology Assessment.

Erin Harvey, David Jackson, Eileen Wang, Enrico Heffler, G. Walter Canonica, John Busby, Pearlanne Zelarney, Sirena Concetta and You Sook Cho declare no relevant conflicts of interest concerning this paper.

## REGABS18011: Withdrawn

## REGABS18012: Understanding the reasons behind self-selecting medications for allergic rhinitis in the community pharmacy

### Sinthia Bosnic-Anticevich^1,2^, Rachel Tan^1^, Biljana Cvetkovski^1^, Vicky Kritikos^1^, David Price ^3,4^, Kwok Yan^1.5^ and Peter Smith^6^.

#### ^1^Quality Use of Respiratory Medicine Group, Woolcock Institute of Medical Research, University of Sydney, Sydney, Australia; ^2^Sydney Local Health District, Sydney, Australia; ^3^Academic Primary Care, University of Aberdeen, Aberdeen, United Kingdom; ^4^Observational and Pragmatic Research Institute Pte Ltd, Singapore, Singapore; ^5^Royal Prince Alfred Hospital, Sydney, Australia; ^6^Clinical Medicine, Griffith University, Southport, Queensland, Australia

**Background:** Allergic rhinitis (AR) is highly prevalent and more than 50% of people with AR self-medicate with over-the-counter medications in the community pharmacy, without seeking professional advice. Many patients select suboptimal treatments for their condition, and this increases incidence of developing complications and/or comorbidities. It is unclear what influences a patient’s decision to self-select medications for AR rather than seek professional advice. This study aims to (i) compare the demographics, clinical characteristics and medication selected, between pharmacy customers who choose to self-select and those who interact with a pharmacist when purchasing medication for AR, and (ii) identify the key factors associated with AR patients’ medication self-selection behaviour.

**Method:** A cross-sectional observational study was conducted in a convenience sample of community pharmacies from the Sydney metropolitan area. Data were collected using a researcher administered survey that included: demographics, pattern of AR symptoms, their impact on quality of life (QOL), triggering factors and medication(s) selected. Univariate and multivariate logistic regression was used to identify factors associated with participants’ medication self-selection behaviour.

Of the 296 recruited participants, 202 were identified with AR, of which 67.8% were female, 54.5% were aged > 40 years, 64.9% had a doctor’s diagnosis of AR, and 69.3% self-selected medication(s). Significant differences were noted in AR symptoms, impact of AR on QOL and medication(s) selected between the two groups. Participants who experienced moderate-severe wheeze were more likely (OR 4.047, 95% CI 1.1555-14.188) to self-select medication(s), and those with AR symptoms impacting on their QOL were less likely (OR 0.369, 95% CI 0.188-0.727) to self-select medication(s).

**Conclusion:** Although people with AR who reported an impact on their QOL were more likely to consult a pharmacist, however the high incidence of self-selection of OTC treatments for AR symptoms in community pharmacy does not reflect the severity of the condition experienced by patients. This indicate that there are also people with AR underestimate the severity of their symptoms and subsequently do not see the need to consult a pharmacist. Nonetheless, on top of having AR, participants who were also experiencing wheeze, were less likely to consult a pharmacist. Pharmacists must be aware of this finding especially in light of the recent “Thunderstorm Asthma” event resulting in serious exacerbations and even death. Pharmacists should alert them regarding these co-existing conditions and provide them with proper education.

**Disclosures:** Sinthia Bosnic-Anticevich**:** Conflict of interests: A member of the Teva Pharmaceuticals Devices International Key Experts Panel; received research support from Research in Real Life; payment for lectures/speaking engagements and for developing educational presentations from Teva and Mundipharma; received Honoria from AstraZeneca, Boehringer Ingelheim, GlaxoSmithKline, for her contribution to advisory boards/key international expert forum. Rachel Tan: Conflict of interests: Nothing to disclose. Biljana Cvetkovski: Conflict of interests: Nothing to disclose**.** Vicky Kritikos: Conflict of interests: Received honoraria from AstraZeneca, GlaxoSmithKline and Pfizer. Kwok Yan: Conflict of interests: Received honoraria for speaking and consulting from AstraZeneca, Boehringer Ingelheim, GlaxoSmithKline, Meda, Mundipharma and Pfizer. Peter Smith: Conflict of interests: A clinical allergist with research and clinical interest in rhinology and has also been a speaker for Meda, GlaxoSmithKline, Novartis, Mundipharma and AstraZeneca. David Price: Conflict of interests: A board membership with Aerocrine, Amgen, AstraZeneca, Boehringer Ingelheim, Chiesi, Meda, Mundipharma, Napp, Novartis, and Teva Pharmaceuticals; consultancy agreements with Almirall, Amgen, AstraZeneca, Boehringer Ingelheim, Chiesi, GlaxoSmithKline, Meda, Mundipharma, Napp, Novartis, Pfizer, Teva Pharmaceuticals, and Theravance; grants and unrestricted funding for investigator-initiated studies (conducted through Observational and Pragmatic Research Institute Pte Ltd) from UK National Health Service, British Lung Foundation, Aerocrine, AKL Research and Development Ltd, AstraZeneca, Boehringer Ingelheim, Chiesi, Meda, Mundipharma, Napp, Novartis, Pfizer, Respiratory Effectiveness Group, Takeda, Teva Pharmaceuticals, Zentiva, and Theravance; payment for lectures/speaking engagements from Almirall, AstraZeneca, Boehringer Ingelheim, Chiesi, Cipla, GlaxoSmithKline, Kyorin, Meda, Merck, Mundipharma, Novartis, Pfizer, Skyepharma, Takeda, and Teva Pharmaceuticals; payment for manuscript preparation from Mundipharma and Teva Pharmaceuticals; payment for the development of educational materials from Novartis and Mundipharma; payment for travel/accommodation/meeting expenses from Aerocrine, Boehringer Ingelheim, Mundipharma, Napp, Novartis, Teva Pharmaceuticals, and AstraZeneca; funding for patient enrolment or completion of research from Chiesi, Teva Pharmaceuticals, Zentiva, and Novartis; stock/stock options from AKL Research and Development Ltd, which produces phytopharmaceuticals; owns 74% of the social enterprise Optimum Patient Care Ltd, UK, and 74% of Observational and Pragmatic Research Institute Pte Ltd, Singapore; and is peer reviewer for grant committees of the Medical Research Council, Efficacy and Mechanism Evaluation programme, and Health Technology Assessment.

## REGABS18013: Withdrawn

## REGABS18014: Withdrawn

## REGABS18015: Asthma patients perspectives on medication; do they need something more than the blue one?

### Alan Kaplan

#### University of Toronto, Toronto, Canada

**Background:** To understand patient perspectives on Asthma medications

**Method:** 20 patients interviewed (first quarter 2016) in seven 90-minute focus groups, four in English(Toronto) and three in French(Montreal). Patient inclusion were those prescribed a regular controller, either ICS monotherapy or combination LABA/ICS.

**Results:** A number of different themes emerged. Asthma was described as “airway closing" in terms of symptoms and only rarely was there a mention of inflammation. They understood the 'blue one' immediately relieved their symptoms, that the preventer was a steroid and that combination puffers contained both. An interesting analogy was the preventer being like an antidepressant to be used to prevent 'sliding back' into depression.

There were many concerns about ICS including safety and becoming dependent on them; an analogy given was like the eyes getting weaker when one depended on their glasses. Many participants only believed that the asthma was present only when there were symptoms. Conversely, they held little hope of being able to live 'free and clear' of the illness and that they must live with a lesser quality of life and a permanent physical ailment.

With education, patients could understand the concept of the SMART strategy and could be taught that a B2 agonist such as Ventolin is a 'band aid solution' with and ICS/LABA such as Symbicort being able to 'do more for me'. Those that were unfamiliar with SMART suggested that they would inquire about it with their physicians.

**Conclusion:** While Asthma is an inflammatory disease requiring regular anti-inflammatory treatment, patients have fears about using ICS and seem to be more comfortable using a single inhaler that can.

**Disclosures:** funded by Astra Zeneca

## REGABS18016: Withdrawn

## REGABS18017: Characterization of community practice COPD by blood eosinophils

### Ronald J. Dandurand

#### CIUSSS de l’Ouest-de-l’Île-de-Montréal, Montreal Chest Institute, Meakins-Christie Labs, Oscillometry Unit, Centre for Innovative Medicine, McGill University Health Centre and Research Institute, Montreal, QC, Canada.

**Background:** The peripheral blood eosinophil count (EC) has been proposed as a biomarker to identify an inhaled corticosteroids responsive COPD phenotype. We wished to determine the prevalence of elevated ECs in community practice COPD and explore what subject parameters, if any, might be associated.

**Method:** 100 subjects with COPD, >10 pack-years of smoking and either FEV_1_/FVC<0.7 (GOLD COPD), or MMEF<65%predicted (non-GOLD COPD), had EC, patient reported outcomes (PRO; CAT, mMRC, AER, chronic bronchitis questionnaire) and PFTs obtained within the same year, and had undergone oscillometry (OS) and quantitative CT scanning (qCT). The mean EC was determined by GOLD2017 Stage and Grade. A frequency distribution histogram of EC was constructed and 3 cut points chosen to establish 4 EC groups by which to compare data. Any parameter suggesting a relationship to EC was subjected to ANOVA. The same 3 cut points were applied singly, resulting in 3 pairs of subject groupings of Hi and Lo EC. Students t-test was then applied to detect differences in parameters means if a difference had been detected by AVOVA.

**Results:** The PFT-OS interval was 9±9 mean±SD months, and PFT-CT, 14±14. Distribution of mean EC by GOLD2017 Stages and Grades was (Stage/Grade, n, mean EC); non-GOLD, 4, 125 cells/μL; I, 26, 225: II, 47, 225; III, 16, 169; IV. 7, 286, and non-GOLD, 4, 125; A, 13, 255; B, 5, 120; C, 48, 225; D, 30, 214 respectively. The frequency distribution of EC is shown in Figure 1.

Cut points of ≥200, ≥300 and ≥400 cells/μL resulted in elevated EC prevalence of 60, 36 and 13% respectively. Of all the biometric, PRO, PFT, OS and qCT parameters (data not shown) only the D_Lco_ appeared to differ by EC groups (ANOVA p=0.035). Dividing subjects into 2 groups using a cut point ≥400 resulted in significant differences in mean D_Lco_ (Hi vs. Lo; 13, 17±2 n, mean±SE mmHg/L/min vs. 87, 13±1, p=0.003) while means did not differ using cut points of either ≥200 or ≥300 (60, 13±1 vs. 40, 13±1 and 36, 14±1 vs. 64, 13±1).

**Conclusion:** Of biometric, PRO, PFT, OS and qCT parameters, only D_Lco_ demonstrated an association with elevated EC. Whether this represents a chance finding due to the number of parameters surveyed or, indeed, reflects a relative preservation of the D_Lco_ suggesting a more asthma-like phenotype of COPD, requires confirmation in larger independent populations.

**Disclosures:** Nothing to disclose.

**Fig. 1 (abstract REGABS18017).**
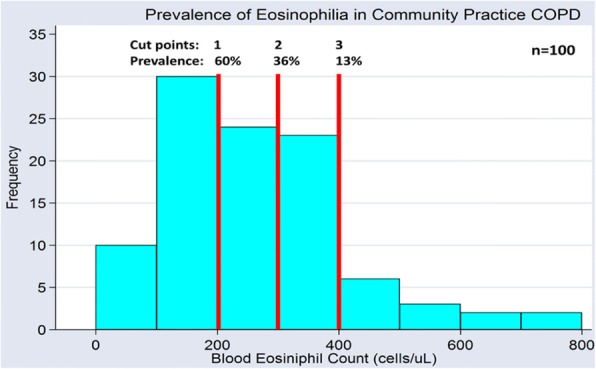
See text for description

## REGABS18018: The effectiveness of the addition of antibiotics to the usual care in the management of asthma exacerbations

### Nikolaos Papadopoulos^1^, Clare Murray^2^, Sarah Lucas^3^, Wanda Phipatanakul^4^, Steve Turner^5^, Alan Kaplan^6^, James Paton^7^, Teoh Oon Hoe^8^, Alberto Papi^9^, John Blakey^10^, Mike Thomas^11^, David Price^12^, Emilio Pizzichini^13^.

#### ^1^Division of Infection, Immunity & Respiratory Medicine, University of Manchester, Manchester, UK and Allergy Research Centre, 2nd Pediatric Clinic, National Kapodistrian, University of Athens, Athens, Greece; ^2^University of Manchester and Royal Manchester Children's Hospital, Manchester, UK; ^3^Respiratory Effectiveness Group, Cromer, UK; ^4^Boston Children's Hospital, Boston, MA, USA; ^5^Child Health, University of Aberdeen, Aberdeen, UK; ^6^Family Physician Airways Group of Canada and Department of Family and Community Medicine, University of Toronto, Toronto, Ontario, Canada; ^7^Child Health, University of Glasgow, Glasgow, UK; ^8^Department of Paediatrics, KK Women's and Children's Hospital, Singapore, Singapore; ^9^Research Center on Asthma and COPD, University of Ferrara, Ferrara, Italy; ^10^Health Services Research, University of Liverpool, Liverpool UK; ^11^Primary Care and Population Sciences, Faculty of Medicine, University of Southampton, Southampton, UK; ^12^Observational and Pragmatic Research Institute, Singapore, Optimum Patient Care, Cambridge, UK, and Academic Centre of Primary Care, University of Aberdeen, Aberdeen, UK; ^13^Department of Medicine, Federal University of Santa Catarina, Santa Catarina, Brazil.

**Background:** Asthma exacerbations are major contributors to asthma morbidity and mortality, and their management presents a major clinical need that is not adequately met by current approaches. Asthma exacerbations are usually managed with bronchodilators and systemic steroids. There is increasing evidence that bacterial infections may contribute to exacerbation severity, with recent randomised controlled trials suggesting that antibiotics may be beneficial in managing exacerbations. These findings warrant further exploration in larger more representative routine care populations.

**Method:** Using retrospective electronic medical records from the Optimum Patient Care Research Database, we have conducted a comparative effectiveness study into managing asthma exacerbations with oral corticosteroids (OCS) alone versus oral corticosteroids plus antibiotics in paediatric and adult asthma populations (Figure 1).

**Results:** The prescribing of OCS alone or OCS plus antibiotics was not a random event. Antibiotics seem to be more commonly prescribed in those who appeared to have more severe asthma, were smokers, and during the winter, presumably due to more infections. Therefore, the groups were matched on GINA treatment step, smoking status, season of index prescription date (IPD) and number of asthma/wheeze consultations in the baseline period.

In the 12-week outcome period survival analysis showed the time to the next asthma/wheeze consultation was increased in adults, but not children, who received an OCS plus antibiotic (hazard ratio 0.94 (95% CI 0.91, 0.96, p<0.001) compared to OCS alone at IPD (Figure 2). In the same period the time to the next asthma/wheeze consultation that resulted in an OCS with or without antibiotic prescription was decreased in adults (p=0.002) and 13-18 yr olds (p=0.046), but not younger children, who received an OCS plus antibiotic (hazard ratios 1.08 (95% CI 1.03, 1.13), 1.23 (95% CI 1.003, 1.50), respectively), compared to OCS alone at IPD.

The time to first hospitalisation or A&E attendance for a lower respiratory complaint were no different in those who received OCS plus antibiotics compared to OCS alone at IPD, during the 12-week outcome period.

**Conclusion:** The addition of antibiotics to oral steroids in the management of asthma exacerbations does have a small effect on reducing subsequent asthma/wheeze consultations, in adults, but more work is required to draw firm conclusions from this data.

**Disclosures:** Nothing to disclose.

**Fig. 1 (abstract REGABS18018).**
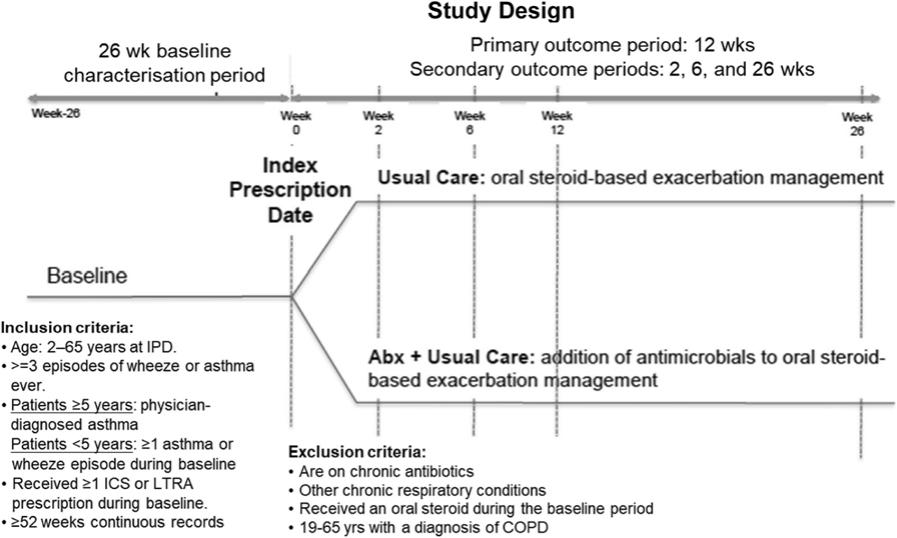
Study Design

**Fig. 2 (abstract REGABS18018).**
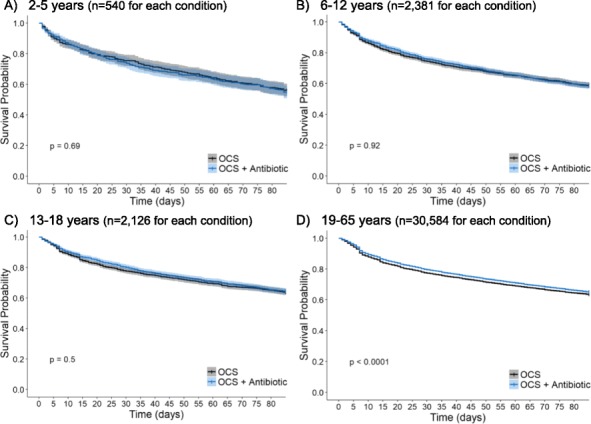
Survival analysis to 1^st^ primary care consultation for asthma/wheeze following IPD. Shading indicates the 95% CI, p-values are calculated from log-rank tests

## REGABS18019: Management of community acquired pneumonia in primary care in the United Kingdom

### Naomi Launders^1^, Chris Winchester^2^, Dermot Ryan^1^, Anjan Nibber^3^, David Price^4^

#### ^1^Respiratory Effectiveness Group, Cambridge, UK; ^2^Oxford PharmaGenesis, Oxford, UK; ^3^University of Oxford Medical School, Oxford, UK; ^4^University of Aberdeen, Aberdeen, UK

**Background:** Community acquired pneumonia (CAP) is a leading infectious cause of mortality in the UK. In 2014, CRB65 scores (a composite score based on: confusion, raised respiratory rate, low blood pressure, age 65 or more) were incorporated into the National Institute for Health & Care Excellence (NICE) pneumonia guidelines^1^. Those with a score of 0 were deemed suitable for management at home, while consideration of hospital-based care was advised for all other patients, particularly those with a score of more than 2. The aim of this study was to describe the diagnosis and management of CAP in primary-care and to evaluate the extent to which the NICE guidelines have been implemented.

**Method:** An observational study was performed using the Optimum Patient Care Research Database (OPCRD), a UK primary-care database containing Electronic Medical Records (EMR) from 4 million patients. Episodes of CAP (>180 days after any prior episode) in adults (≥18 years) between 1 January 2009 and 31 December 2016 and with at least 28 days of continuous EMR before and after diagnosis date were included. As this study focused on patients managed in primary care, episodes of CAP were excluded if no antibiotic prescriptions were recorded, the patients were referred to secondary care, or the diagnosis was retrospective.

**Results:** There were 3,181 episodes of CAP from 3,067 patients in the study period. The incidence of CAP managed in primary care was 18.4 (95% CI: 17.8-19.0) episodes per 100,000 patients in the OPCRD database per year. In 383 episodes (12.0%), at least one consultation for a lower respiratory tract infection (LRTI) was recorded in the 28 days prior to CAP diagnosis, of which 298 (77.8%) were prescribed antibiotics. Consultations for CAP or LRTI in the 28 days following diagnosis were recorded for 950 episodes (29.9%).

CRB65 scores were recorded in six episodes (0.2%) and no episode had all components recorded individually. Recording of CRB65 and CRB65 components improved over time (Figure 1). The most commonly recorded observations and assessments on diagnosis date were pulse rate (289, 9.1%) and chest examination (248, 7.8%; Table 1). The most commonly prescribed antibiotic class at diagnosis was penicillin (1,959; 61.6%), of which 1,422 (72.8%) were amoxicillin.

**Conclusion:** Recording of CRB65 and its components in CAP patients managed in primary care is low. The usefulness of CRB65 scores in primary care needs further investigation.

**Disclosures:** Chris Winchester is an employee, Director and shareholder of Oxford PharmaGenesis and a Director of Oxford Health Policy Forum CIC. Dermot Ryan, David Price and Naomi Launders have no conflicts of interest to disclose.

References:

1. NICE. Pneumonia in adults: diagnosis and management. 2014:https://www.nice.org.uk/guidance/cg191.

**Fig. 1 (abstract REGABS18019).**
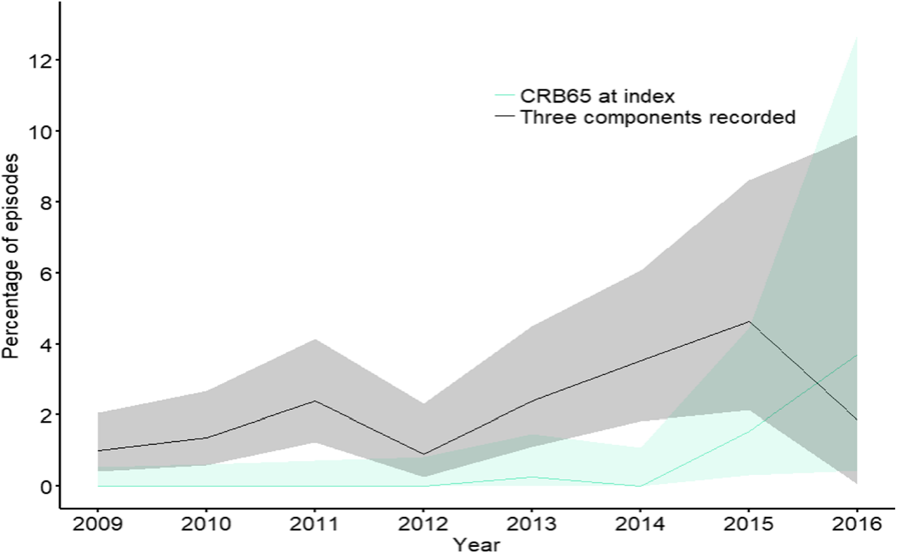
Annual trends in percentage recording of CRB65 scores at index. Where shading is binomial 95% Confidence intervals

**Table 1 (abstract REGABS18019).** Observations and assessments performed on the day of diagnosis

		Number of episodes, n(%)
Blood tests	RBC/WBC/FBC	91 (2.9)
	Liver function	89 (2.8)
	Platelets	84 (2.6)
	Haemoglobin	82 (2.6)
	Protein	68 (2.1)
	C-reactive protein	55 (1.7)
	Glucose	27 (0.8)
	None of the above	22 (0.7)
	Total	150 (4.7)
Observed symptoms	Fever	187 (5.9)
	Cough	182 (5.7)
	Breathlessness	76 (2.4)
	Wheeze	35 (1.1)
	Sputum	32 (1.0)
	Total	416 (13.1)
Investigations	Pulse rate	289 (9.1)
	Chest exam	248 (7.8)
	Oxygen saturation	234 (7.4)
	Spirometry	110 (3.5)
	Tests for organisms (incl. blood tests)	47 (1.5)
	Heart exam	42 (1.3)
	Urine sample	34 (1.1)
	Total	680 (21.4)
Other	Diagnosed organism	346 (10.9)
	Medication review	163 (5.1)
	Smoking advice	60 (1.9)
	Total	532 (16.7)
None of the above		1,868 (58.7)

## REGABS18020: The effects of obesity, weight loss and gastro-oesophageal reflux disease on asthma

### Naomi Launders^1^, Celeste Porsberg^2^, Nicolas Roche^3^, Omar Usmani^4^, Therese Lapperre^2^ on behalf of the Respiratory Effectiveness Group Small Airways working group

#### ^1^Respiratory Effectiveness Group, Cambridge, UK; ^2^ Bispebjerg og Frederiksberg Hospital, Copenhagen, Denmark; ^3^Hôpital Cochin, Paris, France; ^4^National Heart and Lung Institute (NHLI), Imperial College London & Royal Brompton Hospital, London, UK

**Background:** While asthma and obesity frequently occur together, the exact relationship between the two is unclear, as are the mechanisms involved. Poor diet, physical inactivity, gastro-oesophageal reflux disease (GERD), excess adipose tissue and immunological factors related to obesity may all act independently to induce or exacerbate asthma symptoms. Conversely, weight loss through diet or weight loss surgery, may improve asthma symptoms. This study aims to investigate the relationship between asthma symptoms and initial BMI, weight loss, and weight loss method; with consideration of the role that co-morbid GERD plays in modifying the effects of these relationships.

**Method:** The study will consist of two retrospective cohort studies using the Optimum Patient Care Research Database (OPCRD). OPCRD is a large primary care database comprising almost 4.5 million patients from over 600 UK general practices. Both phases of the study will examine small airway function, asthma exacerbations and medications, and asthma-related quality of life.

Phase I will compare asthma outcomes in obese and non-obese adult asthma patients with and without GERD in the five years following asthma diagnosis, and examine the relationship between BMI, GERD and asthma control. Patients with a diagnosis of chronic obstructive pulmonary disease, cancer or aged over 65 will be excluded.

Phase II will examine the effects of weight loss in obese adult asthmatic patients. Asthma outcomes will be compared in obese patients in the five years following with weight loss through diet or weight loss through surgery. Asthma symptoms in those showing no significant weight loss will also be examined. The exact amount of weight loss required will be determined in phase I. Patients with multiple bariatric surgeries or surgeries to remove gastric bands will be excluded.

In both phases, sub-analyses of severe asthma, asthma phenotypes, and obesity class will be performed.

**Results:** Results will be published in a peer reviewed journal.

**Conclusion:** This study will provide valuable information on the effect that obesity and GERD have on asthma symptoms, and the role that weight loss, through diet or surgery, plays in improving symptoms. Results from this study will be used to inform future studies on the role of extra-fine vs. fine inhaled corticosteroids in obese asthmatic patients, the effects of GERD surgery on asthma and the effect of obesity, GERD and weight loss on asthma in children.

**Disclosures:** Naomi Launders, Celeste Porsberg, Omar Usmani and Therese Lapperre have no conflicts of interest to disclose. Dr. Roche: Reports and personal fees from Boehringer Ingelheim, Novartis, Pfizer and personal fees from Teva, GSK, AstraZeneca, Chiesi, Mundipharma, Cipla, Sanofi, Sandoz, 3M, Zambon.

## REGABS18021: Database studies in uganda- an opportunity for global research where chronic lung disease matters

### Rupert Jones^1,2^, Bruce Kirenga ^2^, Winceslas Katagira^2^, Andrew Barton^1^, Trishul Siddharthan^3^, William Checkley^3^

#### ^1^Peninsula School of Medicine, University of Plymouth, Plymouth, UK; ^2^Makerere Lung Institute, College of Health Sciences, Kampala, Uganda; ^3^Division of Pulmonary and Critical Care, School of Medicine, Johns Hopkins University, Baltimore, MD, USA

**Background:** the WHO estimates that COPD is now the third leading cause of death globally and most deaths occur in developing countries. COPD prevalence rose 35% 10 years in Africa through the combination of tobacco smoking, indoor and outdoor air-pollution, tuberculosis and HIV in a deprived population. The phenotypes seen are different to those in developed countries and include restrictive disorders such as fibrosis and lung destruction. Little is known of the causes and natural history of these disorders.

**Aim:** To outline epidemiological approaches undertaken, planned and potential to address chronic lung disease in Africa

**Method:** Description of resources of existing datasets come from cross-sectional surveys, longitudinal cohort and other trials.

**Results**: Many existing datasets with high quality data in Uganda have potential to address current disease burden for example, post TB lung damage in the WHO TB survey. Multinational projects provide opportunities to compare the risk factors and impacts of lung disease between countries. Forward planning will allow the data being collected in trials and clinical practice to answer new questions: (i) Extending cross-sectional surveys to longitudinal cohorts with appropriate items will answer questions such as do obstructive lung disease progress? What are predictors of progression? (ii) adding new items in existing cohorts for example in URAC project there is potential to examine microbiome in sputum, genetic markers while tracking disease progression is relatively easy in Uganda.

**Conclusion**: The neglected pandemic of chronic lung disease in Africa may be addressed by using existing resources and relatively small investments to generate and optimise longitudinal datasets.

**Disclosures:** Nothing to disclose.

**Table 1 (abstract REGABS18021).** Existing datasets with high quality data in Uganda

	Population	Design	Items
WHO TB Uganda National Survey	40,000 national representative sample	Cross-sectional	Respiratory symptoms, status of TB, smoking, HIV, Chest XRay
Uganda Registry Asthma and COPD	(i)Approximately 500 COPD, 500 asthma and 200 Post TB patients (ii) 420 COPD patients in 7 centre	Longitudinal, 6 monthly visits	Spirometry, health status (CCQ), biometrics, exacerbation frequency
Uganda National Asthma Survey	3,400 participants over 12year old in 5 districts	Cross-sectional	ISAAC, WHO survey 1 &2, Cardiovascular risk
Africa Severe Asthma programme	Uganda (826), Kenya, (425), Ethiopia (425). 1676 in total	Cross-sectional	Spirometry, reversibility, questionnaires. Exhaled nitric oxide, blood eosinophils, stool microscopy
Link survey and FRESH AIR Uganda	1600 rural participants 1000 Urban (Kampala)	Cross-sectional	Spirometry, symptoms, risk factors biomass and smoking

## REGABS18022: Changes in asthma drug use in France between 2006 and 2015: a claims data study

### Manon Belhassen^1,2^, Maëva Nolin^1^, Marine Ginoux^1^, Eric Van Ganse^1,2,3^

#### PELyon, Lyon, France; ^2^EA 7425 HESPER, Université Claude-Bernard-Lyon1, Lyon, France; ^3^Service de Pneumologie, Hôpital de la Croix Rousse, Hospices Civils de Lyon, Lyon, France

**Background**: Data are needed to understand the trends of asthma drug use over time. The objective was to describe the use of asthma controller therapy between 2006 and 2015 in France.

**Method**: Repeated database analyses were conducted each year from 2006 to 2015, using the EGB (Echantillon Généraliste de Bénéficiaires, 1/97e random sample of the French national claims database SNIIRAM). Adult patients aged 18-40, without Long Term Disease status and/or hospital admissions related to COPD and with at least one dispensation of inhaled corticosteroids (IC), Long-Acting Beta2 Agonist (LABA) or Fixed Dose Combination of LABA/IC (FDC) were identified. Four specific profiles were investigated: single IC without LABA (IC group), single LABA without IC (LABA group), single IC and LABA in separate canisters (IC+LABA group), FDC without dispensation of IC nor LABA (FDC group). Each year, patients’ characteristics, and percentage of patients in each group were described.

**Results**: The overall percentage of asthma controller therapy users remained stable between 2006 and 2015 (1.5% of the EGB population). Mean age (31.3 to 31.0 years) and gender (60.8% to 61.9% females) varied little. Hospitalizations for asthma (0.6% to 0.5%) were rare. Patients with ≥1 annual dispensation of oral corticosteroids increased from 44.0% in 2006 to 50.5% in 2015. Between 2006 and 2015, percentage of patients decreased in the LABA group (4.1% to 2.0%), in the LABA+IC group (6.2% to 2.0%), and in the IC group (44.8% to 39.1%) whereas it increased in the FDC group (44.8% to 56.8%).

**Conclusion**: In France, prevalence of asthma controller therapy use remained stable between 2006 and 2015 but FDC tended to replace other asthma medications. Use of oral corticosteroids increased during the period.

**Disclosures:** Nothing to disclose.

## REGABS18023: RELEVANT: a tool for quality appraisal of observational comparative effectiveness research

### Nicolas Roche^1^, Naomi Launders^2^, Jon Campbell^3^ on behalf of the REG-EAACI Taskforce on quality standards in asthma comparative effectiveness research

#### Hôpital Cochin, Paris, France; ^2^Respiratory Effectiveness Group, Cambridge, UK; ^3^Center for Pharmaceutical Outcomes Research, University of Colorado Denver, Denver, Colorado, US

**Background:** Randomised controlled trials (RCTs) are required to establish the efficacy and safety of novel asthma treatments, but pragmatic randomized trials and observational comparative effectiveness research (CER) studies are also necessary to test the external validity of their findings. While quality standards for RCTs are well-defined, e.g., by the CONSORT statements, this is less so in the field of observational comparative effectiveness research. To address this, the Respiratory Effectiveness Group (REG) and European Academy of Allergy and Clinical immunology (EAACI) convened a joint Task Force to set and test quality standards for observational CER in asthma, leading to the development of the Real Life Evidence AssessmeNt Tool (RELEVANT).

**Method:** A systematic review of previously published quality checklists was performed to identify criteria to include in RELEVANT. The initial tool was reviewed by the Task Force and the central recommendation was to reduce the number of criteria minimize inter-rater variability. The tool was pilot tested by subgroups of the task force, followed by an extended pilot of 22 participants identified through an open invitation to all REG collaborators. Finally, an online version was created using Google forms. RELEVANT was then used to appraise studies relating to four selected PICOT questions: the influence of adherence, smoking, inhaler device and particle size on asthma treatment effectiveness.

**Results:** RELEVANT comprises primary and secondary criteria. Any failure of an article to meet primary criteria precludes the study’s use to support a guideline. Inter-rater agreement of the initial tool was variable across individual criteria (33-100%), but following iterative feedback, inter-rater agreement in the extended pilot was greater than 70% for 94% of primary criteria and 93% of secondary criteria. The final tool consists of 21 sub-items across seven domains: Background, Design, Measures, Analysis, Results, Discussion/Interpretation, Conflicts of Interest. A total of 46 articles relating to the four PICOT questions were identified and assessed using RELEVANT. For all PICOT questions, assessed observational studies yielded results with possible impact on clinical practice in areas where similar evidence from RCTs is lacking.

**Conclusion:** RELEVANT is the first quality checklist to assist in the appraisal of published observational CER developed through iterative feedback derived from pilot implementation and inter-rater agreement evaluation. Further steps now need to be discussed to determine how the tool can be disseminated and its use implemented in collaboration with scientific societies, guidelines developers and other stakeholders.

**Disclosures:** Dr. Campbell has received consultancy or research grants over the past three years from: Agency for Healthcare Research and Quality, ALSAM Foundation, Amgen, AstraZeneca, Bayer, Biogen Idec., Boehringer Ingelheim, Center for Disease Control and Prevention, Colorado Medicaid, Creative Group Inc., Enterprise Community Partners Inc., Institute for Clinical and Economic Review, Kaiser Permanente, Mallinckrodt, National Institute of Health, National Multiple Sclerosis Society, PhRMA Foundation, Precision for Value, Teva, Research in Real Life Ltd., Respiratory Effectiveness Group, and Zogenix Inc. Dr Roche: Reports and personal fees from Boehringer Ingelheim, Novartis, Pfizer and personal fees from Teva, GSK, AstraZeneca, Chiesi, Mundipharma, Cipla, Sanofi, Sandoz, 3M, Zambon.

## REGABS18024: Assessing real-life challenges in paediatric asthma

### Alexander G. Mathioudakis^1^, Ioana Agache^2^, Leonard Bacharier^3^, Jose Castro-Rodriguez^4^, Adnan Custovic^5^, Antoine Deschildre^6^, Zuzana Diamant^7^, Francine M. Ducharme^8^, James E Gern^9^, Gunilla Hedlin^10^, Elham M. Hossny^11^, Tuomas Jartti^12^, Alan Kaplan^13^, Robert F. Lemanske^9^, Peter Le Souef^14^, Mika J Makela^15^, Paolo M. Matricardi^16^, Mário Morais-Almeida^17^, Antonio Nieto Garcia^18^, Wanda Phipatanakul^19^, Paulo MC Pitrez^20^, David Price^21^, Petr Pohunek^22^, Peter D. Sly^23^, Steve Turner^24^, Gary Wong^25^, Heather Zar^26^, Nikolaos G. Papadopoulos^1^

#### Division of Infection, Immunity and Respiratory Medicine, The University of Manchester, Manchester, UK; ^2^ Faculty of Medicine, Transylvania University, Brasov, Romania; ^3^ Division of Allergy, Immunology, and Pulmonary Medicine, Department of Pediatrics, Washington University, St. Louis, MO, USA; ^4^ Department of Pediatrics, School of Medicine, Pontifical Universidad Catolica de Chile, Santiago, Chile; ^5^ Department of Paediatrics, Imperial College London, London, UK; ^6^ Center of Infection and Immunity of Lille, University of Lille, Lille, France; ^7^ Department of Respiratory Medicine and Allergology, Institute of Clinical Science, Skåne University Hospital, Lund, Belgium; ^8^ Department of Pediatrics, University of Montreal, Montreal, Canada; ^9^ Department of Pediatrics and Medicine, University of Wisconsin School of Medicine and Public Health, Madison, WI, USA; ^10^ Department of Women’s and Children’s Health, Karolinska Institutet, Stockholm, Sweden; ^11^ Pediatric Allergy and Immunology Unit, Children’s Hospital, Ain Shams University, Cairo, Egypt; ^12^ Department of Paediatrics, Turku University Hospital and University of Turku, Turku, Finland; ^13^Family Physician Airways Group of Canada, University of Toronto, Toronto, Ontario, Canada; ^14^ School of Paediatrics and Child Health, University of Western Australia, Perth, Australia; ^15^ Helsinki University Skin and Allergy Hospital and University of Helsinki, Helsinki, Finland; ^16^ AG Molecular Allergology and Immunomodulation, Department of Pediatric Pneumology and Immunology, Charité Medical University, Berlin, Germany; ^17^ Allergy and Clinical Immunology Department, Hospital CUF-Descobertas, Lisboa, Portugal; ^18^ Pediatric Allergy Unity, University Hospital La Fe, Valencia, Spain; ^19^ Children's Hospital Boston, Pediatric Allergy and Immunology, Boston, Massachusetts, USA; ^20^ Laboratory of Respiratory Physiology, Infant Center, School of Medicine, Pontifícia Universidade Católica do Rio Grande do Sul (PUCRS), Porto Alegre, Brazil; ^21^ Academic Centre of Primary Care, University of Aberdeen, Aberdeen, UK; ^22^ Paediatric Dept, 2nd Faculty of Medicine, Charles University and Motol University Hospital, Prague, Czech Republic; ^23^ Child Health Research Centre, University of Queensland, Queensland, Australia; ^24^ Child Health, Royal Aberdeen Children’s Hospital and University of Aberdeen, Aberdeen, UK; ^25^ Department of Paediatrics, Faculty of Medicine, The Chinese University of Hong Kong, Sha Tin, Hong Kong; ^26^ Department of Paediatrics and Child Health, Red Cross Children's Hospital and Medical Research Council Unit on Child and Adolescent Health, University of Cape Town, Cape Town, South Africa.

**Background:** Paediatric asthma is a common, chronic lower respiratory disease, associated with significant morbidity and socio-economical burden. While it is beyond doubt that asthma in childhood represents a distinct entity, with diverging underlying mechanisms and triggers, outcomes and response to treatments, it is still approached as an extension of adult asthma in clinical practice. Characteristically, treatment recommendations are mostly based on indirect evidence from trials in adults and the available definitions still fail to address the particularities of paediatric asthma. The objective of this project is to identify and prioritize unmet research and policy needs in paediatric asthma.

**Method:** We conducted a comprehensive online open-question qualitative survey that was targeted to world-leading experts, clinical researchers in the field of paediatric asthma, enquiring on unanswered clinically-relevant questions around paediatric asthma and specifically around its definition, classification, natural history, diagnosis, assessment, drug therapy and non-drug management, monitoring, management of exacerbations or other clinically relevant questions.

**Results:** The survey was completed by 22 international experts (80% of those invited), from 16 countries and 5 continents. The numerous highlighted unanswered questions collectively confirm unmet needs in all aspects of the clinical approach to paediatric asthma. Primarily, there is an urgent need for the development of consensus on the definition, criteria for diagnosis and classification of paediatric asthma, which is anticipated to differ from adult asthma and possibly among different age groups. Moreover, characterization of clinical phenotypes and identification of predictors of disease severity, persistence and responsiveness to treatment needs also represent unanswered clinical questions. Evidence syntheses and new direct evidence is required to guide all aspects of pharmacological (including biological factors) and non-pharmacological treatment of stable paediatric asthma and exacerbations. Asthma prevention, adherence to treatment, inhaler technique, (tele)monitoring, community (school) interventions and the role of early routine assessment of comorbidities were additional areas of uncertainty. Surprisingly, the responses did not considerably differ from a similar exercise conducted 10 years ago.

**Conclusion:** Many clinical questions related to paediatric asthma remain unanswered, as a result of the disease’s complexity, the limitations in conducting clinical research in children and the subsequent significant shortcoming of good-quality evidence in all domains assessed. There is a need for an international paediatric asthma network aiming to promote high-quality clinical research, the development of an international paediatric asthma registry and evidence-based recommendations.

**Disclosures:** Nothing to disclose.

## REGABS18025: Severe asthma databases: a global comparison

### Nevaashni Eleangovan^1^, Lakmini Bulathsinhala^1^, Naeimeh Hosseini^1^, Victoria Carter^1^, Liam G. Heaney^2^, Andrew Menzies-Gow^3^, Peter G. Gibson^4,5^, Matthew Peters^6^, Chin Kook Rhee^7^, Celeste Porsbjerg^8^, Roland Buhl^9^, Anke H. Maitland-van der Zee^10^, G. Walter Canonica^11^, Eileen Wang^12^, Luis Perez de Llano^13^, Borja G. Cosio^14^, Richard Costello^15^, David Price^1,16,17^

#### Optimum Patient Care, Cambridge, UK; ^2^UK Severe Asthma Registry, Queen’s University Belfast, Belfast, Northern Ireland; ^3^UK Severe Asthma Registry, Royal Brompton & Harefield NHS Foundation Trust, London, UK; ^4^Australasian Severe Asthma Network, Priority Research Centre for Healthy Lungs, University of Newcastle, Newcastle, Australia; ^5^Hunter Medical Research Institute, Department of Respiratory and Sleep Medicine, John Hunter Hospital, New Lambton Heights, Australia; ^6^University of Sydney Medical School, Sydney, Australia; ^7^Catholic University of Korea, Seoul, South Korea; ^8^Bispebjerg Hospital, Copenhagen University, Copenhagen, Denmark; ^9^Mainz University Hospital, Mainz, Germany; ^10^University of Amsterdam, Amsterdam, The Netherlands; ^11^Personalized Medicine Asthma & Allergy Clinic, Humanitas University & Research Hospital, Milan, Italy; ^12^Division of Allergy and Clinical Immunology, National Jewish Health, Denver, Colorado, USA; ^13^Spanish Society of Pulmonology and Thoracic Surgery, Madrid, Spain; ^14^Son Espases University Hospita-IdISBa-Ciberes, Mallorca, Spain; ^15^RCSI Royal College of Surgeons in Ireland, Dublin, Ireland; ^16^Observational and Pragmatic Research Institute, Singapore, Singapore; ^17^Academic Primary Care, University of Aberdeen, Aberdeen, UK

**Background:** Severe asthma is a heterogenous disease with varying clinical manifestations. Several registries have been developed globally to study the natural history of this disease. However, few studies have compared the information collected by such registries. The aim of this study was to compare the data fields currently captured by severe asthma registries across the globe.

**Method:** Medline, EMBASE, Web of Science, web searches as well as consultations with leaders of severe asthma research databases were used to identify severe asthma repositories. Investigators were contacted to collect information on data collection specifications. Data dictionaries from respective databases were used for systematic comparison and pooling of variables. A database of data fields (as indicator variables) and countries (as rows or elements of analysis) was created. Categories of variables, such as demographics and diagnostics, were used for ease of reporting, and Stata14 and Microsoft Excel were used to organize and tabulate data fields.

**Results:** From the eighteen identified databases, data collection specifications from a total of ten severe asthma research repositories, covering 255 sites globally, were received. All country-specific databases collect information on asthma medications as per Global Initiative for Asthma guidelines for severe asthma. Among the non-asthma medications, anti-histamine data was most prevalently collected (seven repositories). With the emergence of novel asthma medications, the Spanish, Netherlands, German and Italian registries collected medication safety information. Sputum eosinophil, blood eosinophil and IgE levels were collected by all research databases, reaffirming the pivotal role these tests play in management of severe asthma. For assessing asthma control, seven used the Asthma Control Test, six used the GINA Asthma Control, and four used the Asthma Control Questionnaire. The approach for ascertaining adherence for inhaled and/or oral corticosteroids differed across countries. The United Kingdom, Australia, the Netherlands, Ireland, Nordics and USA use objective methods such as prescription records and/or blood cortisol levels, while Spain and South Korea use subjective compliance questions (two do not collect data on adherence).

**Conclusion:** Severe asthma databases across the globe converge on collecting similar data field categories, while they differ significantly on the specific data fields included. A standard list of variables captured across countries will increase the statistical power of future studies by allowing for data interoperability.

**Disclosures:** Nevaashni Eleangovan, Lakmini Bulathsinhala, Naeimeh Hosseini and Victoria Carter are employees of Optimum Patient Care.

Liam Heaney has taken part in advisory boards and given lectures at meetings supported by GlaxoSmithKline, Respivert, Merck Sharpe & Dohme, Nycomed, Boehringer Ingelheim, Teva, Vectura, Novartis and AstraZeneca. He declares sponsorship for attending international scientific meetings from AstraZeneca, Boehringer Ingelheim, GlaxoSmithKline and Napp Pharmaceuticals; and speaker fees from AstraZeneca, Aerocrine, Hoffman la Roche and Teva.

Andrew Menzies-Gow declares grants from AstraZeneca, Boehringer Ingelheim, GlaxoSmithKline and Hoffman La Roche; consultancy agreements with AstraZeneca; personal fees from AstraZeneca, Boehringer Ingelheim, GlaxoSmithKline, Hoffman La Roche, Napp Pharmaceuticals, Novartis, Teva and Vectura; non-financial support from AstraZeneca, Boehringer Ingelheim and Napp Pharmaceuticals; and payment for travel expenses to international conferences from AstraZeneca, Boehringer Ingelheim and Napp Pharmaceuticals.

Peter Gibson declares grants from AstraZeneca, GlaxoSmithKline and Novartis; and personal fees from AstraZeneca and GlaxoSmithKline.

Matthew Peters declares personal fees and non-financial support from AstraZeneca and GlaxoSmithKline.

Chin Kook Rhee declares consultancy and lecture fees from AstraZeneca, Boehringer Ingelheim, GlaxoSmithKline, MSD, Mundipharma, Novartis, Sandoz, Takeda and Teva-Handok.

Roland Buhl declares personal fees from AstraZeneca, Boehringer Ingelheim, Chiesi, Novartis, Roche and Teva, as well as grants to Mainz University from Boehringer Ingelheim, GlaxoSmithKline, Novartis and Roche.

Anke H. Maitland-van der Zee declares unrestricted grants from GlaxoSmithKline and personal fees for advisory board activities from AstraZeneca.

Luis Perez de Llano declares grants from AstraZeneca, Chiesi and Teva; personal fees from AstraZeneca, Boehringer Ingelheim, Chiesi, Esteve, GlaxoSmithKline, Mundipharma, Novartis, Sanofi and Teva; non-financial support from Boehringer Ingelheim, Esteve, GlaxoSmithKline, Menarini, Mundipharma, Novartis and Teva.

Borja G Cosio declares grants from Chiesi; personal fees for advisory board activities from Chiesi and AstraZeneca; and payment for lectures/speaking engagements from Chiesi, Novartis, Menarini and AstraZeneca.

Richard Costello is named on the INCA device patent application; PCT/EP2013/067932, filed on the 29th of August 2013.

David Price declares board membership with Aerocrine, Amgen, AstraZeneca, Boehringer Ingelheim, Chiesi, Mylan, Mundipharma, Napp Pharmaceuticals, Novartis and Teva; consultancy agreements with Almirall, Amgen, AstraZeneca, Boehringer Ingelheim, Chiesi, GlaxoSmithKline, Mylan, Mundipharma, Napp Pharmaceuticals, Novartis, Pfizer, Teva and Theravance; grants and unrestricted funding for investigator-initiated studies (conducted through Observational and Pragmatic Research Institute Pte Ltd) from Aerocrine, AKL Research and Development Ltd, AstraZeneca, Boehringer Ingelheim, British Lung Foundation, Chiesi, Mylan, Mundipharma, Napp Pharmaceuticals, Novartis, Pfizer, Respiratory Effectiveness Group, Teva, Theravance, UK National Health Service and Zentiva; payment for lectures/speaking engagements from Almirall, AstraZeneca, Boehringer Ingelheim, Chiesi, Cipla, GlaxoSmithKline, Kyorin, Mylan, Merck, Mundipharma, Novartis, Pfizer, Skyepharma and Teva; payment for manuscript preparation from Mundipharma and Teva; payment for the development of educational materials from Mundipharma and Novartis; payment for travel/accommodation/meeting expenses from Aerocrine, AstraZeneca, Boehringer Ingelheim, Mundipharma, Napp Pharmaceuticals, Novartis and Teva; funding for patient enrolment or completion of research from Chiesi, Novartis, Teva and Zentiva; stock/stock options from AKL Research and Development Ltd which produces phytopharmaceuticals; owns 74% of the social enterprise Optimum Patient Care Ltd (Australia, Singapore, and UK) and 74% of Observational and Pragmatic Research Institute Pte Ltd (Singapore); and is peer reviewer for grant committees of the Efficacy and Mechanism Evaluation programme and Health Technology Assessment.

Celeste Porsbjerg, Eileen Wang and G.Walter Canonica declare no relevant conflicts of interest concerning this abstract.

## REGABS18026: Oscillometry upper limit of normal at World COPD Day 2017

### Ronald J. Dandurand^1,2,3,4^, Ryan Nantal-Smith^1,2^, Matthew Shorofsky^1^, Stephanie Alexandre^1^, Sarah Cadet^1^, Jessica Ghaleb^1^, Tara Glover^1^, Chantal Forget^1^, Belhadj Mohamed^1^ and Zoltan Hantos^5^

#### CIUSSS de l’Ouest-de-l’Île-de-Montréal, Montreal, Canada; ^2^Montreal Chest Institute, Montreal, Canada; ^3^Meakins-Christie Labs, Montreal, Canada; ^4^Oscillometry Unit, Centre for Innovative Medicine, McGill University Health Centre and Research Institute, Montreal, QC, Canada; and ^5^Szeged University, Szeged, Hungary

**Background:** Oscillometry is a fast and easy form of objective lung function measurement whose deployment is limited, in part, due to a lack of normative data. We wished to establish an upper limit of normal (ULN) for the integrated area of low frequency reactance (A_X_), the oscillometry estimate of ventilatory inhomogeneity, by a random sampling of visitors to a community hospital open house marking World COPD Day 2017.

**Method:** A COPD nurse asked random visitors to participate, obtained written informed consent, administered a respiratory health survey and the patient reported outcomes (PRO) CAT, mMRC, ACT and chronic bronchitis questionnaire. Subjects completed first oscillometry (tremoFlo C-100, Thorasys, Montreal, Canada) and then spirometry (microLab, Vyaire, Hochberg, Germany) by respiratory therapists respecting ATS/ERS criteria. Subject data were divided into groups of those with and without a history of respiratory disease. Group differences were tested with Bonferroni corrected Student’s t-tests or χ^2^, as appropriate for normally distributed data, and Mann-Whitney U tests for non-normally distributed data. The A_X_ ULN was calculated using the mean+2SDs of ln transformed A_X_ after exclusion of outliers amongst those without a history of respiratory disease. Associations between the FEV_1_, lnA_X_ and PROs were measured using Bonferroni corrected Pearson correlations. The study had IRB approval.

**Results:** 47 subjects completed the study; 11 with (9 asthma, 2 COPD) and 36 without a history of respiratory disease. Groups didn’t differ in terms of age, sex distribution, smoking history but did for ACT, FEV_1_%predicted, FEV_1_/FVC, MMEF %predicted, resistance at 5 Hz (R_5_), reactance at 5 Hz (X_5_), frequency dependence of resistance (R_5-20_), and A_X_ (Table 1).

The lnA_X_ frequency histogram demonstrated a bimodal distribution (Figure 1).

A_X_ correlated with CAT and mMRC more strongly than FEV_1_ (r=0.53, p=0.001 vs. r=-0.41, p=0.041 and r=0.48, p=0.006 vs. r=-0.44, p=0.021, respectively).

**Conclusion:** Both spirometry and oscillometry demonstrated significant differences between subjects with and without a history of respiratory disease. Oscillometry correlated more strongly with PROs than spirometry. A_X_ demonstrated a bimodal distribution amongst subjects without a history of respiratory disease as we have seen previously in 3 independent populations. The A_X_ ULN is similar to our previous observations suggesting a disease defining threshold of 10-20 cmH_2_0/L.

**Future Directions:** All subjects with A_X_≥8 cmH_2_O/L are to undergo formal respiratory consultation, imaging and complete pulmonary function testing permitting estimation of negative and positive predictive values for this A_X_ ULN.

**Disclosures:** Nothing to disclose.

**Table 1 (abstract REGABS18026).** Biometrics, Patient Reported Outcomes, Spirometry and Oscillometry

Biometrics p/χ^2^	Healthy Group, n=36			Disease Group, n=11			
Age (mean years ± sem)	50	±	3	50	±	5	NS
Sex (M : F)	9	:	27	2	:	9	NS
BMI (kg/m^2^)	26	±	1	30	±	3	NS
Smoking History							
Pack-Years	6	±	2	9	±	5	NS
Current : Ex	12	:	24	4	:	7	NS
Patient Reported Outcomes							
COPD Assessment Test (CAT)	6.22	±	1.05	12.64	±	2.90	NS
mMRC Dyspnea Scale (mMRC)	0.25	±	0.07	0.73	±	0.27	NS
Asthma Control Test (ACT)	0.67	±	0.30	5.00	±	1.70	<0.005
Chronic bronchitis (Yes : No)	2	:	34	2	:	9	NS
Spirometry							
FEV_1_ (L)	3.00	±	0.21	2.24	±	0.15	NS
FEV_1_ (% predicted)	106	±	3	83	±	4	<0.005
FVC (L)	3.74	±	0.24	3.19	±	0.18	NS
FVC (% predicted)	112	±	3	100	±	6	NS
FEV_1_/FVC (%)	80	±	1	71	±	4	<0.05
MMEF (L/s)	2.92	±	0.25	1.76	±	0.26	NS
MMEF (% predicted)	81	±	4	52	±	7	<0.05
Oscillometry							
R_5_ (median (IRQ) cmH_2_O/L/s)	3.64	(2.82, 4.25)		4.79	(4.05, 0.87)		<0.05
R_5-20_ (cmH_2_O/L/s)	0.39	(0.14, 0.78)		1.10	(0.80, 1.70)		<0.05
X_5_ (cmH_2_O/L/s)	-1.44	(-1.72, -1.14)		-2.04	(-3.28, -1.20)		<0.05
F_res_ (Hz)	16	(13, 20)		25	(21, 28)		NS
A_X_ (cmH_2_O/L)	6	(5, 11)		16	(13, 28)		<0.001

**Fig. 1 (abstract REGABS18026).**
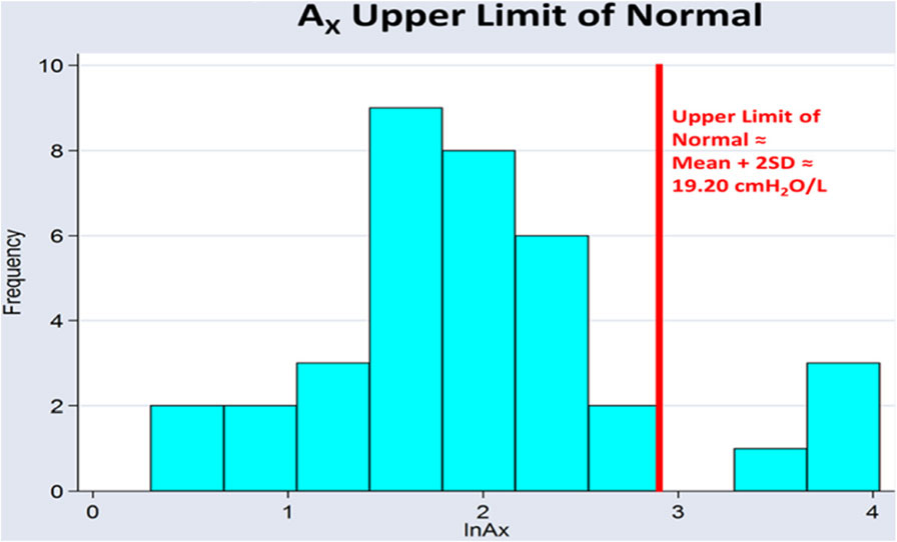
See text description

## REGABS18027: Smoking cessation therapies among hospitalized surgical patients with COPD

### Mihaela S Stefan, Meng-Shiou Shieh, Penelope Pekow, Pack Quinn, Steven Bernstein, Peter K Lindenauer

#### Institute for Healthcare Delivery and Population Science; University of Massachusetts Medical School-Baystate, Springfield, MA, US

**Background:** Smoking in the perioperative period has been associated with increased postoperative complications, hospital costs and health care resources. Tobacco cessation has been prioritized as a quality improvement performance measure by the Joint Commission and in recent American College of Surgeons position statement. Hospitalization for surgery and the period of forced cessation that it involves is a ‘window of opportunity’ for delivering smoking cessation interventions. No studies have yet investigated the utilization of smoking cessation pharmacotherapy (SCP) in surgical patients. Given the lack of data, we sought to describe the prescription of SCP in smokers with a COPD diagnosis undergoing a surgical procedure.

**Method:** Retrospective study using a large database of 466 hospitals in the US. We included adults 18 years of age or older, admitted from January to December 2016 for an elective or urgent surgery with an estimated length of stay of more than 2 days. We restricted the analysis to active smokers with a diagnosis of COPD (defined based on the ICD-10 codes). Primary outcome was utilization of any SCP: varenicline tartrate, and nicotine replacement therapy, including nicotine patch, gum, lozenge, and inhaler. We collected demographics, insurance and comorbidities data and classified the surgeries in urgent versus emergent and into eleven main categories. We also identified oncological diagnosis possibly related to smoking.

We developed a hierarchical generalized linear model to determine independent patient, surgery and hospital factors associated with the use of SCP. We also describe variation in SCP use across hospitals with 10 or more eligible patients and calculated the median odds ratio (MOR) to quantify the influence of the hospital on the probability of receiving SCP.

**Results:** Among 43,708 surgical patients with a diagnosis of active smoking and COPD only 1805 patients (4.1%) had SCP administered at least once during their hospital stay and the nicotine patch was the predominant mode (91.5%). Compared with patients who did not receive SCP, those who received SCP were younger: mean age 67.5 vs 60.5 years and more likely to have Medicaid insurance. They were more likely to have psychiatric diagnoses (OR: 1.5;95% CI: 1.3,1.7), alcohol disorders (OR: 2.5; 95% CI: 2.1, 3.0), drug abuse disorders (OR: 1.7; 95% CI: 1.4, 2.0) or oncological diagnoses (OR: 1.4; 95% CI: 1.1, 1.6) but were less likely to have diabetes, obesity (OR: 0.6; 95% CI: 0.5, 0.7) or renal failure. Patients undergoing urgent surgeries were also more likely to have SCP prescribed (OR: 1.4; 95% CI: 1.3, 1.6). The median hospital rate of SCP use was 3.4%; IQR: 1.2, 6.5%, and hospitals located in the South, non-teaching and rural hospitals had higher rates of use. The MOR was 1.2; 95% CI: 1.1, 1.3.

**Conclusion:** Despite strong evidence that smoking is associated with surgical complications and the potential benefit to initiating therapy during hospitalization, we found that SCP is rarely used in the postoperative period in patients with active smoking and with a COPD diagnosis. Further studies should determine strategies to implement perioperative smoking cessation with outpatient follow-up to improve quit rates and subsequently patients outcomes.

**Disclosures:** Nothing to disclose.

## REGABS18028: Assessing the availability, functionality, utility and acceptance of smart inhalers

### Naomi Launders^1^, John Blakey^2^, David Price^3^, Omar Usmani^4^ on behalf of the Respiratory Effectiveness Group Technologies working group

#### Respiratory Effectiveness Group, Cambridge, UK; ^2^Royal Liverpool Hospital, Liverpool, UK; ^3^University of Aberdeen, Aberdeen, UK; ^4^National Heart and Lung Institute (NHLI), Imperial College London & Royal Brompton Hospital, London, UK

**Background:** While inhaler therapies are the mainstay of treatment for both asthma and Chronic Obstructive Pulmonary Disease, adherence to these medications is often sub-optimal. Reduced adherence increases the risk of exacerbations and hospitalisations and worsen prognoses. Monitoring of dosage, technique and adherence through “smart inhalers” offers an opportunity to provide patient specific services to improve adherence. Despite the availability of a plethora of smart inhaler products, there is a paucity of evidence on the feasibility and user acceptability of specific systems, both from the viewpoint of patients and of physicians.

This study will provide an overview of all smart inhalers currently available, and those coming to market soon and whether they meet the key needs of respiratory physicians and other health care professionals.

**Method:** Systematic review: PubMed will be searched using the following search terms; ((“electronic” OR “monitoring” OR “sensing” OR “digital” OR “smart” OR “mhealth”) AND “inhaler”); with no language restrictions in place. Studies evaluating, describing or using inhalers with a digital component will be included in the analysis. Studies using devices that are no longer available will be excluded.

Keywords and inhaler names will be recorded and, if applicable, will be included in a second round of searching. Reference lists of review articles will be used to identify additional studies. The studies will be reviewed independently by two researchers and disagreements discussed and referred to a third researcher where necessary. Data on the inhaler type, the technology used, data capture and access, and opinions of the user will be extracted for analysis.

Delphi exercise: An electronic Delphi panel will be constructed, consisting of primary and secondary care physicians, nurses, pharmacists and physiotherapists. Members will be recruited through professional contacts and networks. The Delphi process will comprise of three rounds. Round one will contain open-ended questions. Round two will contain of key themes gathered from round one, with importance of these themes rated on a Likert scale. Round three will include refined statements, with agreement measured on a multi-point scale.

**Results:** The results of this study will be communicated in a peer-reviewed journal.

**Conclusion:** This study will review the current availability, functionality, utility and acceptability of smart inhalers. It will be used to inform further research into the use of technologies to predict and detect exacerbations, and to optimise adherence and therefore patient outcomes.

**Disclosures:** Nothing to disclose.

## REGABS18029: A desktop helper for asthma management

### Alan Kaplan^1^, Ioanna Tsiggliani^2^, Miguel Roman-Rodriguez^3^, David Price^4^

#### IPCRG Committee, Toronto, Canada; ^2^IPCRG Committee, Crete, Greece; ^3^IPCRG Committee, Baleares, Spain; and ^4^IPCRG Committee, Paya Lebar, Singapore.

**Background:** Inhaled Corticosteroids (ICS) have been overprescribed in the management of COPD. Research has highlighted that ICS are not benign and have significant side effects and that adequate bronchodilation may be as good/better for exacerbation prevention in many patients. Patients with concomitant COPD and Asthma require treatment with ICS, but they represent a minority of all COPD patients. Removal of ICS would benefit many patients but requires a systematic approach to ensure the correct patients are considered and withdrawal is done safely.

**Method:** Many studies have revealed that there is over-prescription of ICS in GOLD A and B patients that runs contrary to current recommendations. Additionally, ICS combinations are often started at early stages of the disease when benefits may be negligible. Newly available LABA/LAMA medications have demonstrated excellent symptom and exacerbation benefits, providing ICS free pharmacotherapy for COPD management in appropriate patients.

A desktop helper has been created for international dissemination by a group of primary care respiratory interested clinicians to educate, guide, and support clinicians with ICS withdrawal in appropriate COPD patients after a literature review and consensus decision making process.

**Results:** The Desktop helper is available in the tools section of the International Primary Care Respiratory Group

**Conclusion:** ICS are overused in COPD. This desktop helper will give clinicians an approach to if, how and when to reduce this use in clinical daily practice.

**Disclosures:** This work was supported by Novartis Global.

## REGABS18030: Adherence to inhaled corticosteroids and asthma control in UK primary care records: what happens between prescription events?

### Alexandra Dima^1^, Marcia Vervloet^2^, Patrick Souverein^3^, on behalf on the REG Adherence Working Group. Gene Colice^4^, Eric van Ganse^5^, Hilary Pinnock^6^, Iain Small^7^, Cynthia Rand^8^, Michelle Eakin^8^, Janet Holbrook^9^, Miguel Román Rodríguez^10^, Nemr Eid^11^, Randall Brown^12^ & David Price^13^

#### Lyon Pharmaco-Epidemiology Unit-HESPER EA 7425-Claude Bernard Lyon 1 University, Lyon, France; ^*2*^NIVEL, Netherlands Institute for Health Services Research, Utrecht, The Netherlands; ^3^Division of Pharmacoepidemiology and Clinical Pharmacology, Utrecht Institute for Pharmaceutical Sciences, Utrecht University, Utrecht, The Netherlands.

**Background:** In asthma care adherence to inhaled corticosteroids (ICS) is usually suboptimal, which is associated with increased individual and societal asthma burden. Electronic healthcare records allow investigation of the role of ICS adherence in long-term routine care, however most evidence to date is cross-sectional, has limited granularity, does not distinguish between stages of adherence and between sequential and simultaneous associations. This project aimed to address prior methodological limitations by focusing on the relationships between ICS implementation and asthma control measured between each two consecutive prescription events.

**Method:** A retrospective observational study was conducted using UK primary care records from the Optimum Patient Care Research Database. The index prescription date (IPD) was the date of the first ICS prescription, the baseline period was one year prior to IPD and the follow-up period 2 years post IPD. Inclusion criteria were: physician-diagnosed asthma, age ≥6 years, ≥2 ICS and/or SABA prescriptions in each follow-up year, and no LABA, LTRA or maintenance oral corticosteroids during the baseline year. ICS adherence (implementation) and risk domain asthma control (RDAC; no exacerbations, antibiotics or outpatient visits) were computed for each period between two prescriptions (prescription interval) (Figure 1).

Multilevel analyses examined to what extent (1) the variation in RDAC can be explained by implementation rates in the same or preceding interval, and (2) implementation can be explained by simultaneous and preceding RDAC (or RDAC components), controlling for socio-demographic and clinical characteristics.

**Results:** The dataset contained 94,498 prescription intervals; 14,425 intervals with 0% adherence and 55,971 intervals with 100% adherence were excluded, leaving 24,102 intervals (4.0-99.6% adherence) from 10,472 patients. Implementation within the same interval had a small positive association with RDAC, and several patient characteristics were found to influence RDAC (Table 1). Overusing SABA, having ≥1 antibiotic prescription, and asthma-related outpatient visits in the same interval had a negative influence on implementation (Table 1). Overusing SABA in the previous interval was associated with lower implementation in the current interval.

**Conclusion:** A weak reciprocal association may reflect slightly lower implementation in prescription intervals which include events indicative of loss of control. The lack of an association between implementation and control in consecutive intervals may suggest that patients adapt their use of medication to their current needs without this impacting on their subsequent control; similarly, current asthma control does not influence patient’s medication use in a next prescription interval.

**Disclosures:** Nothing to disclose.

**Fig. 1 (abstract REGABS18030).**
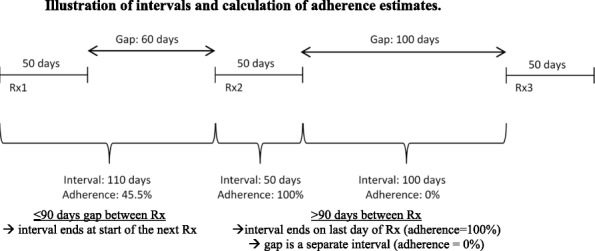
Illustration of intervals and calculation of adherence estimates

**Table 1 (abstract REGABS18030).** Results from the multilevel analyses. Only significant predictors shown

Hypothesis 1, dependent variable RDAC	Model 1	
	OR (95% CI)	
Characteristics interval level		
Adherence within interval	1.01 (1.00-1.01)^**^	
Characteristics patient level		
Gender (ref=female)	1.58 (1.34-1.87)^***^	
Smoking history (ref=current)		
none	1.47 (1.15-1.89)^**^	
former	1.61 (1.18-2.19)^**^	
Diagnosed with (ref=no)		
COPD	0.52 (0.35-0.78)^**^	
CCI (ref=low (≤4))	0.65 (0.50-0.85)^**^	
Doses in the device	1.00 (0.99-1.00)^*^	
Daily dose	1.07 (1.00-1.15)^*^	
The following predictors were found to be non-significant. **Characteristics interval level:** SABA overuse within interval/lagged, adherence lagged. **Characteristics patient level:** age, BMI, deprivation, asthma duration, type of ICS device, diagnosed with- rhinitis, allergic rhinitis, hayfever, GERD, other respiratory disease.		
Hypothesis 2, dependent variable adherence	Model 1^a^	Model 2^b^
	Estimate (SE)	Estimate (SE)
Characteristics interval level		
*Within same interval*		
≥1 Prescription of antibiotics *(ref=no)*	-1.77 (0.64)^**^	
≥1 Asthma-related outpatient visits (ref=no)	-2.32 (1.17)^*^	
SABA overuse (ref=no)	-6.68 (0.42)^***^	-6.69 (0.42)^***^
Risk domain asthma control (ref=no)		2.18 (0.46)^**^
*Previous interval*		
SABA overuse (ref=no)	-1.22 (0.44)^**^	-1.21 (0.44)^**^
Characteristics patient level		
Age	0.07 (0.01)^***^	0.07 (0.01)^***^
Deprivation (ref=Q1 most affluent)		
Q2	0.94 (0.88)	0.91 (0.88)
Q3	2.05 (0.91)^*^	2.02 (0.91)^*^
Q4	0.51 (0.91)	0.46 (0.91)
Q5 (most deprived)	0.96 (0.98)	0.93 (0.98)
Diagnosed with (ref=no)		
Hay fever	-2.63 (0.79)^***^	-2.62 (0.79)^***^
COPD	2.75 (1.10)^*^	2.59 (1.10)^*^
Doses in the device	0.13 (0.0)^***^	0.13 (0.0)^***^
Daily dose	-3.88 (0.15)^***^	-3.87 (0.15)^***^
The following predictors were found to be non-significant. Characteristics interval level, within same interval ≥1: asthma-related hospitalizations, respiratory-related hospitalizations, asthma-related hospitalizations & emergency visits, prescriptions of acute OCS, moderate to severe exacerbations. Characteristics interval level, previous interval ≥1: asthma-related hospitalizations, respiratory-related hospitalizations, asthma-related hospitalizations & emergency visits, prescriptions of acute OCS, prescription of antibiotics, asthma-related outpatient visits, moderate to severe exacerbations, risk domain asthma control. Characteristics patient level: gender, BMI, smoking history, asthma duration, CCI, diagnosed with- rhinitis, allergic rhinitis, GERD, other respiratory disease		
^a^ Separate asthma events, both within the same interval as lagged. ^b^Only composite risk domain asthma control, both within the same interval as lagged. Significance levels: * p<0.05; ** p<0.01; *** p<0.001		

